# Accelerated Recruitment of New Brain Development Genes into the Human Genome

**DOI:** 10.1371/journal.pbio.1001179

**Published:** 2011-10-18

**Authors:** Yong E. Zhang, Patrick Landback, Maria D. Vibranovski, Manyuan Long

**Affiliations:** Department of Ecology and Evolution, The University of Chicago, Chicago, Illinois, United States of America; Trinity College Dublin, Ireland

## Abstract

How the human brain evolved has attracted tremendous interests for decades. Motivated by case studies of primate-specific genes implicated in brain function, we examined whether or not the young genes, those emerging genome-wide in the lineages specific to the primates or rodents, showed distinct spatial and temporal patterns of transcription compared to old genes, which had existed before primate and rodent split. We found consistent patterns across different sources of expression data: there is a significantly larger proportion of young genes expressed in the fetal or infant brain of humans than in mouse, and more young genes in humans have expression biased toward early developing brains than old genes. Most of these young genes are expressed in the evolutionarily newest part of human brain, the neocortex. Remarkably, we also identified a number of human-specific genes which are expressed in the prefrontal cortex, which is implicated in complex cognitive behaviors. The young genes upregulated in the early developing human brain play diverse functional roles, with a significant enrichment of transcription factors. Genes originating from different mechanisms show a similar expression bias in the developing brain. Moreover, we found that the young genes upregulated in early brain development showed rapid protein evolution compared to old genes also expressed in the fetal brain. Strikingly, genes expressed in the neocortex arose soon after its morphological origin. These four lines of evidence suggest that positive selection for brain function may have contributed to the origination of young genes expressed in the developing brain. These data demonstrate a striking recruitment of new genes into the early development of the human brain.

## Abstract 2

Systematic transcriptional profiling across human and mouse revealed that evolutionarily young genes are overrepresented in the developing (fetal and infant) human neocortex.

## Author Summary

The genetic changes that contribute to the evolution of the human brain have always attracted wide interest. There is an emerging consensus that while there have been no major patterns of genome-wide changes to the coding regions of brain-related genes, changes in the regulation of these genes, and especially in the *cis*-regulatory elements that control their transcription, have played a key role. Here, we examined the expression profile of genes in both fetal and adult brains of human and mouse, and discovered an unexpected pattern across different transcriptome profiling platforms. In particular, we found that an excess of young (recently evolved) genes are expressed in the early (fetal or infant) developing human brain compared with those in mouse brain. Expression data covering numerous subregions of the developing brain further demonstrate that these young genes are mainly upregulated in the neocortex. They originated in the evolutionary period during which the neocortex was expanding, suggesting the functional association of new genes with this newly evolving brain structure. Our data reveal that evolutionary change in the development of the human brain happened at the protein level by gene origination and also via evolution of regulatory networks, as intimated by the enrichment of primate-specific transcriptional regulators in our dataset.

## Introduction

For decades, researchers have strove to answer the question of what genetic changes underlie the evolution of the human brain. Evolution in gene regulation was proposed to underlie human uniqueness [1]. Although gene expression in the adult brain appears to be conserved between human and mouse [2], the human brain shows a much higher complexity in fetal development, during which an order of magnitude more alternative transcripts are expressed in human than mouse [3]. Furthermore, numerous studies show that genes expressed in the fetal brain are more often associated with accelerated sequence evolution in their *cis*-regulatory regions compared to the genomic background [4]–[7]. These studies indicate that regulatory changes may contribute to the evolution of the human brain.

On the protein level, a genome-wide study reported that the sequences of proteins involved in the nervous system evolved faster in primates than in rodents [8]. However, slower evolution of the proteins expressed in the primate brain was also observed [9–10]. Other case studies proposed that the microcephaly-associated gene (*ASPM*) and the microcephalin gene (*MCPH1*) had undergone positive selection in the human lineage [11]–[12]. However, criticisms arose over whether the polymorphism patterns of *ASPM* and *MCPH1* in human populations were relevant to positive selection [13]–[14].

These discussions and debates, while interesting, were based on human gene databases where the annotations favored conserved, old genes. However, recent comparative genomic analyses identified a large number of new genes [15]–[16]. For example, many cancer-related domains emerged during the origination of multicellular metazoan organisms [17] and the timing of the gene gain events on the mammalian X chromosome reflects its evolutionary history [18]–[19]. Moreover, there is evidence that some new genes might have brain functions. For example, one protein family (DUF1220) underwent primate-specific expansion and shows high expression in adult human brain [20].

An understanding of the evolution of brain morphology is useful in formulating hypotheses about the molecular evolution of the primate brain. As the outer layer of cerebrum, the neocortex underlies the mental capabilities of humans [21]. It is generally believed to be the evolutionarily latest addition to the brain compared to other regions [21]–[22]. However, whether it originated in the tetrapod ancestor or in the amniote ancestor was debatable [22]. In contrast, non-neocortical regions such as striatum, hippocampus, thalamus, or cerebellum are shared across the vertebrates, or at least all tetrapods [22]–[25]. The neocortex can be divided into subregions, with the prefrontal cortex (PFC) showing the most remarkable expansion in primates, especially in human [21]. Some parts of the PFC, like the orbital PFC, are shared by nonprimate mammals and are responsible for emotional aspects in decision making [22]. Some others are unique to primates, like the lateral PFC which underlies the rational aspects of decision making [22].

In this report, we developed a new approach that correlates the ages of genes with transcription data to detect recent evolution of the human brain. By aligning orthologous syntenic regions across the vertebrate phylogeny, we previously determined in which branch of the mouse or human lineage a new gene arose, providing the age for 90% of all genes in the human and mouse genomes [19]. By combining this dataset with publically available transcriptome data, we observed an unexpected accelerated origination of new genes which are upregulated in the early developmental stages (fetal and infant) of human brains relative to mouse.

## Results

### The Early Brain Development of Humans Recruited Excess New Genes

**Figure 1:**
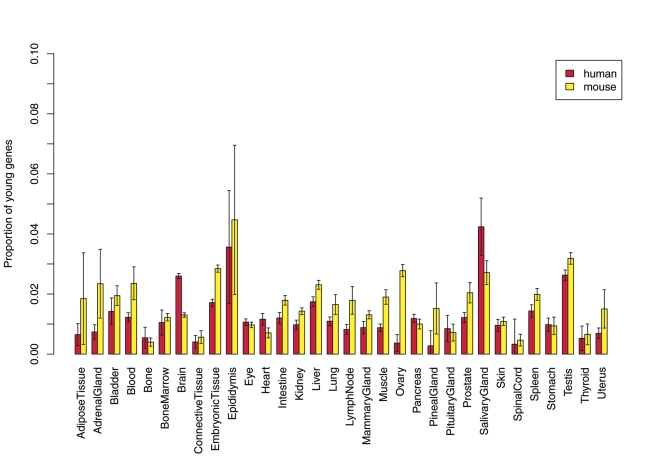
New gene contribution to various tissue transcriptomes. The barplot shows the proportion of young genes out of all genes expressed in tissue or organ categories shared by UniGene human and mouse. For each category, mean and 2-fold standard deviation were plotted, which were generated with 100 bootstrapping replicates of background EST data. Only the brain shows a significant excess of new human genes based on Fisher's Exact Test (FET) with Bonferroni correction.

The UniGene database is a collection of millions of expressed sequence tags (ESTs) taken from thousands of RNA libraries covering dozens of human tissues or organs at different developmental stages [26]. We started by analyzing this comprehensive dataset to characterize the contribution of new genes to the transcriptome of numerous tissues and organs, i.e. to detect how many lineage-specific genes are expressed in a given tissue out of all genes expressed in the same tissue (Materials and Methods). Surprisingly, across dozens of samples, human young genes (primate-specific genes) contribute a significantly larger proportion of all genes expressed in the brain compared to mouse young genes (rodent-specific genes) (408 versus 191 or 3% versus 1.5%, Fisher's Exact Test, FET *p* = 3×10^−13^ after multiple test correction; Figure 1). Such a difference was not due to any ascertainment bias resulting from the fact that the UniGene database has relatively more human brain ESTs (Figure S1). ESTs with developmental stage information further show that human young genes are more often expressed in the fetal brain (175 versus 51 or 2% versus 0.6%, FET *p* = 2×10^−13^), while there is no significant difference between the proportions of young genes expressed in the adult brains of human and mouse (Figure S2). Considering that the UniGene data cover numerous tissues and organs, these observations reveal that the transcriptome of the human fetal brain is significantly enriched with young genes.

Although the UniGene has a high coverage of samples which enables a broad comparison of expression between human and mouse, the coverage of individual genes is often low for a specific sample and it cannot provide quantitative measurement of gene expression. Thus, we took advantage of additional expression data to confirm upregulation of young genes in the fetal brain of humans and investigate which part of the human brain contributes to such a pattern.

**Figure 2:**
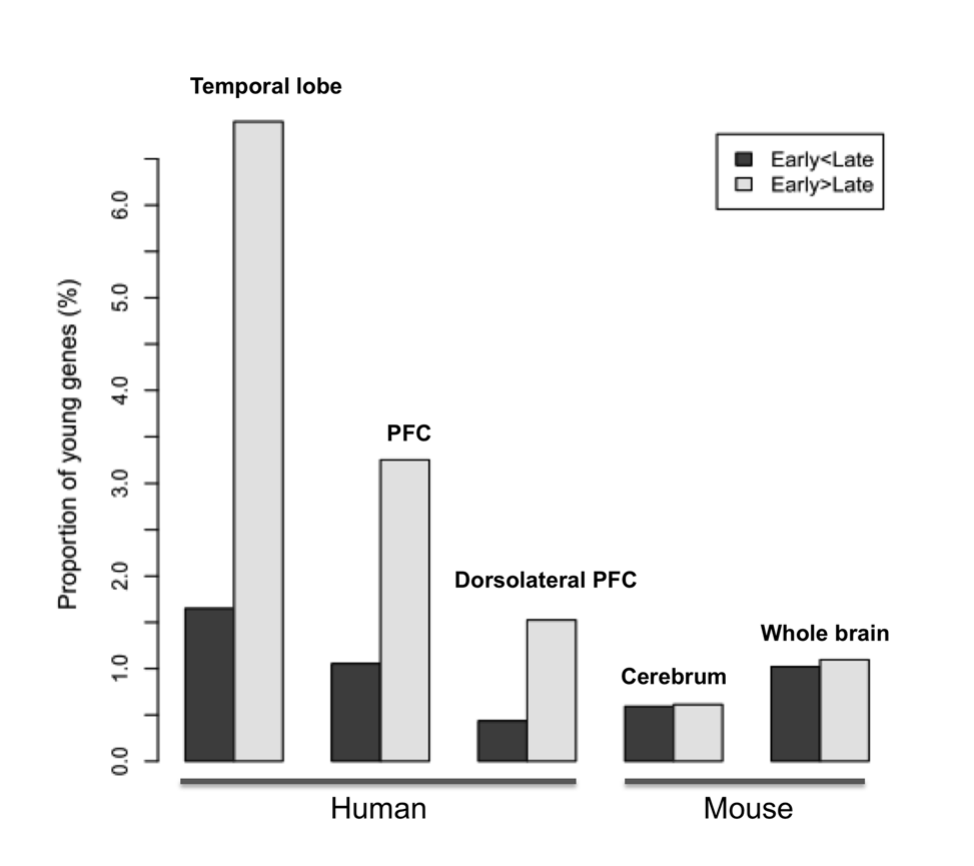
Proportion of young genes out of all genes differentially expressed between developmental stages. For all samples, we compared two developmental stages, identified differentially expressed genes, and then plotted the proportion of young genes out of all early stage or late stage biased genes (Methods). The temporal lobe (one part of the neocortex) and cerebrum data compared fetal and adult brains, while the other three datasets compared infant with subsequent stages (Tables S1, S2).

Exon array profiling of 13 fetal brain regions [4] showed that up to 576 (39%) young genes are upregulated in the neocortex, relative to non-neocortical regions of the brain such as the cerebellum or striatum (Materials and Methods). In contrast, only 10% of young genes are more abundantly expressed in non-neocortical regions. Thus, the expression of young genes in the human fetal brain revealed by EST data is mainly contributed by the neocortex. If these young genes are indeed involved in the development of the neocortex, we expect that their expression would be upregulated in the fetus relative to the adult. Consistent with this prediction, three expression datasets profiling different neocortex regions with various platforms show that young genes are more often upregulated in the fetal or infant brain and much less frequently upregulated in late developing brain (Figure 2, Table S1). Specifically, there are three times as many young genes with predominantly fetal or infant expression. In contrast, old genes predating the primate and rodent split are roughly equally distributed between early and late developing brains (Table S1).

The EST data suggest that this enrichment pattern may be distinct in the human lineage, compared to the mouse. Since the neocortex is relatively small and simple in the mouse brain [21], it is impossible for us to make an exact comparison between human and mouse. However, at least for the cerebrum or whole brain, mouse young genes show similar abundance between different stages (Figure 2, Table S2). Moreover, consistent with the EST data, human young genes contribute significantly more to the set of genes upregulated in early development compared to mouse young genes (1.5%∼7% versus 0.5%∼1%, FET *p*<10^−8^).

One can argue that the higher transcription of young genes in early human development might not be brain-specific, but also true for other organs of the fetus. EST profiling across both human and mouse rejected this possibility, since all fetal tissues except the brain show similar abundance of young genes across fetal and adult life stages in both human and mouse (Figure S3). Another possibility is that many human young genes might be pseudogenes, and thus the pattern does not indicate a biological significance at the level of brain evolution. However, we observed that the evolutionary rates of proteins encoded by new genes were generally lower than the rates at synonymous sites in the same gene sequences (as described in the later section on positive selection), clearly revealing evolutionary constraint on functional genes. Furthermore, after excluding genes without peptide evidence [27], human young genes are still upregulated in fetal brain relative to old genes (FET *p* = 0.002; Table S3). Finally, human young genes do not show a lack of regulatory elements such as insulators or enhancers relative to old genes, suggesting that the majority of these genes are functional (Figure S4).

Given the high coverage of RNA-sequencing (RNA-seq) [28], we subsequently focused on fetal brain biased genes identified by these data (temporal lobe data in Figure 2 and Tables S1, S4) and investigated their function and evolution.

### Young Genes Upregulated in the Fetal Brain Play Diverse Roles

We used the DAVID functional annotations [29] to determine if any functional classes described by Gene Ontology (GO) terms were overrepresented in the fetal brain biased genes, and found a significant enrichment of transcriptional regulators compared to other young genes or fetal brain biased old genes (Table 1). Accelerated emergence of transcription factors (mainly zinc finger proteins, ZNF) accounts for the higher proportion of young transcription factors in humans compared to mouse. Specifically, out of 1,309 human young genes with InterPro domain annotation [30], 176 (13.4%) genes encode transcription factor related domains [31]. This proportion drops to 7.2% in mouse (FET *p* = 8×10^−10^). Together with their fast sequence evolution [32], transcription factors could play an important role during human evolution. For example, *ZNF85* emerged after the split of anthropoid and prosimian primates [19],[33]. Expressional studies showed this adult testis-specific protein represses transcription by binding to DNA in a zinc-dependent way [33]. The RNA-seq data showed that *ZNF85* was expressed significantly higher in the fetal brain relative to the adult brain (Likelihood test *p* = 0, Materials and Methods), suggesting a possible developmental role.

**Table 1:** Over-represented GO terms in fetal brain biased young genes compared to other young genes (a) and fetal brain biased old genes (b).

(a)		
Term	Fold Enrichment	FDR
GO:0006350∼transcription	2.0	6.5E-09
GO:0008270∼zinc ion binding	1.8	1.7E-07
GO:0003677∼DNA binding	1.8	3.0E-07
GO:0043169∼cation binding	1.7	1.6E-06
GO:0046872∼metal ion binding	1.7	1.6E-06
GO:0043167∼ion binding	1.7	1.6E-06
GO:0046914∼transition metal ion binding	1.7	2.1E-06
GO:0045449∼regulation of transcription	1.8	2.6E-06
GO:0051252∼regulation of RNA metabolic process	1.8	1.5E-05
GO:0006355∼regulation of transcription, DNA-dependent	1.8	3.5E-05
GO:0005840∼ribosome	3.4	0.03

(b)		
Term	Fold Enrichment	FDR
GO:0008270∼zinc ion binding	2.1	5.9E-08
GO:0051252∼regulation of RNA metabolic process	2.3	2.4E-07
GO:0006355∼regulation of transcription, DNA-dependent	2.3	3.3E-07
GO:0046914∼transition metal ion binding	1.9	1.3E-06
GO:0006350∼transcription	2.0	3.1E-06
GO:0003677∼DNA binding	1.9	8.3E-06
GO:0045449∼regulation of transcription	1.8	2.1E-04
GO:0046872∼metal ion binding	1.6	5.6E-04
GO:0043169∼cation binding	1.6	6.6E-04
GO:0043167∼ion binding	1.6	9.2E-04
GO:0005840∼ribosome	5.7	1.5E-03
GO:0033279∼ribosomal subunit	6.4	0.02

GO ID together with a short description. Only terms with a False Discovery Rate (FDR) smaller than 0.05 were presented.

Genes lacking GO annotations are neglected by this analysis. One such case is the *morpheus* family, which underwent multiple rounds of duplication in primate linage and showed remarkable protein-level divergences [34]. This family has not been previously associated with any brain functions [35]. However, we found that out of seven young genes belonging to the *morpheus* family, six show upregulation in the fetal brain. Since at least one member of this family was found to be associated with the nuclear pore complex [34], regulation of nuclear pores might be implicated in the early brain development.

### Positive Selection Contributed to the Evolution of Fetal Brain Biased Young Genes

We next investigated the evolutionary mechanisms underlying the origination and subsequent evolution of the fetal brain biased genes. First, we examined whether these genes are generated by relatively few mutational events, e.g. segmental duplications [36], which would violate assumptions of the FET test in Table S1, as the genes are not statistically independent of each other. We found these genes are scattered across the whole genome, demonstrating that they are generated by many independent events (Figure S5). Moreover, based on chromosomal coordinates, we pooled neighboring genes into clusters if they share the same age and transcriptional bias. Given two distance cutoffs (100,000 bases and 1 million bases), young transcriptional clusters continue to be more often expressed in the fetal brain compared to old transcriptional clusters (FET *p*<2.2×10^−16^).

**Figure 3:**
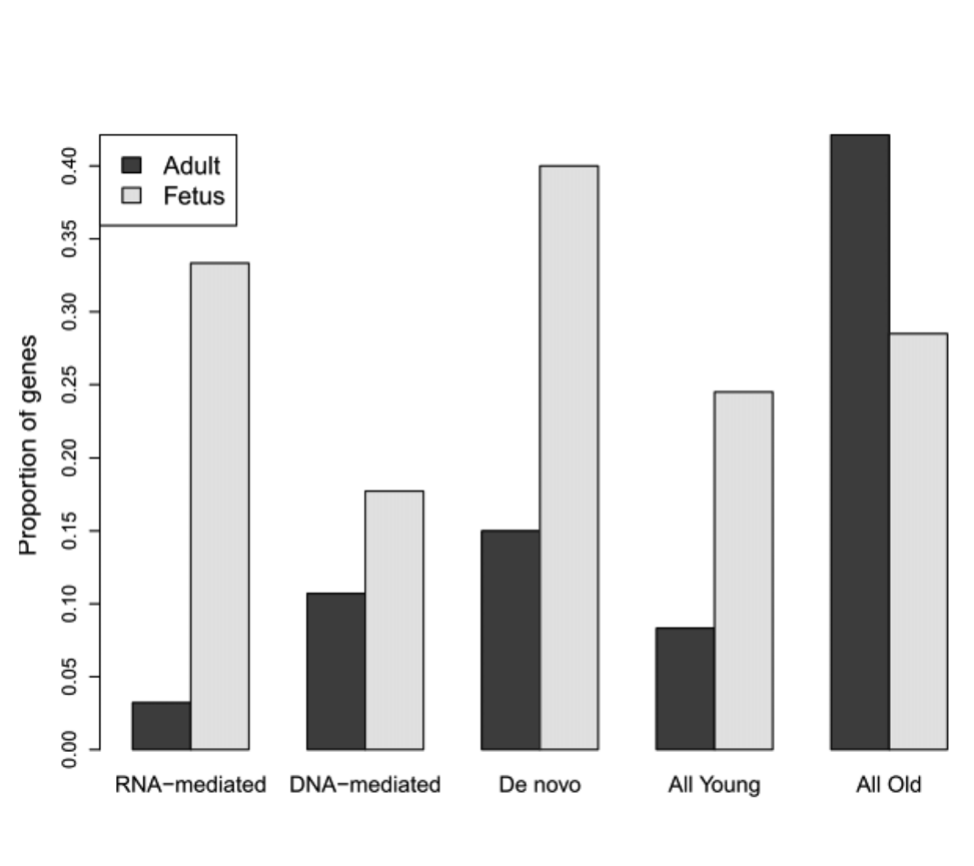
Origination mechanisms of genes up-regulated in the adult and fetal brain. Within each category, the barplot shows the proportion of genes up-regulated in adult brain and in fetal brain, respectively. Binomial test reveals that new genes originated by various mechanisms are significantly more frequently up-regulated in fetal brain (*p*<0.05).

Examination of the gene structure and homology further revealed that these genes were generated by DNA-mediated duplication, RNA-mediated duplication (retroposition), and *de novo* origination (which created a protein without a parental locus) (Figure 3). In other words, young genes created by all major gene origination mechanisms tend to be upregulated in fetal brain. Such generality suggests that a systematic force instead of a mutational bias associated with a specific origination mechanism contributed to the excess of young genes in the fetal brain.

**Figure 4:**
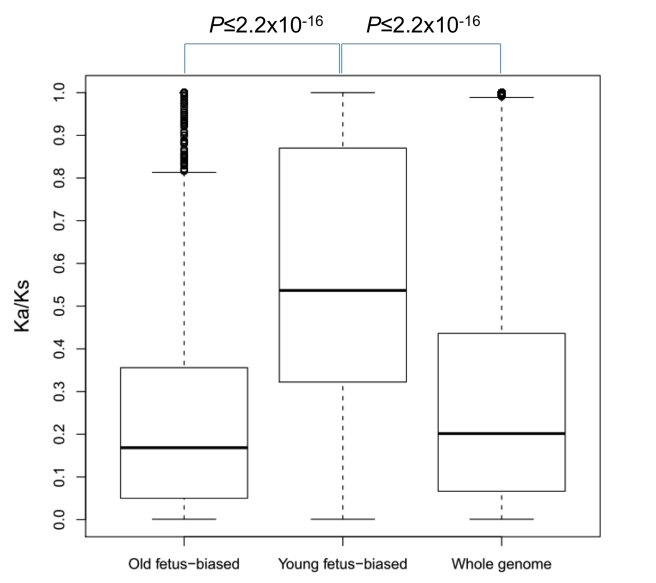
*Ka/Ks* distribution across different group of genes. All *Ka/Ks* values greater than 1 were trimmed to 1.

We further examined the protein evolution rates of these new genes expressed in the fetal brain. We downloaded orthologous coding region alignment between human and chimp from UCSC genome browser [37] and measured the ratio of the nonsynonymous substitutions to synonymous substitutions (*Ka/Ks*, Materials and Methods). As shown in Figure 4, young genes with expression biased towards the fetal brain evolved significantly faster than either old genes with fetal biased expression or the genome-wide average (0.54 versus 0.17 or 0.20, Wilcoxon rank tests *p*≤2.2×10^−16^).

Acceleration of protein evolution could be caused by relaxation of functional constraint or driven by positive selection. Although it is difficult to quantitatively disentangle these two factors, McDonald-Kreitman tests based on human/chimp divergence and human polymorphism data [38]–[39] revealed that positive selection contributes to the fixation of amino-acid substitutions in at least some young fetus-brain biased genes. Specifically, using the genome-wide data generated by this method [39], we identified 16 fetal brain biased genes, and five of these (30%) were subject to positive selection (Table 2). Consistently, we identified a lower proportion of positively selected genes among the old genes upregulated in the fetal brain (14%, FET *p* = 0.06) or the genome-wide average (15%, FET *p* = 0.07) in the set reported in [39].

**Table 2:** Selection intensity on 16 young fetal brain biased genes estimated by McDonald–Kreitman tests with Poisson random field [39].

RefSeq	Symbol	ds	ps	dn	pn	p	u	sd
NM_133473	ZNF431	5	0	11	0	0.00102	8.61813	4.83494
NM_182492	DKFZp434O021	2	0	6	0	0.00814	7.68427	4.90936
NM_145298	APOBEC3F	0	1	11	2	0.0296	4.10728	3.49844
NM_018933	PCDHB13	1	0	2	0	0.06178	6.39172	5.06886
NM_153608	MGC17986	7	0	8	2	0.08628	3.18396	3.28736
NM_033213	MGC12466	0	0	1	0	0.13642	5.36558	5.53891
NM_001700	AZU1	1	3	1	0	0.13726	5.35647	5.58777
NM_024341	ZNF557	2	0	3	1	0.16624	3.58025	4.13862
NM_020880	ZNF530	4	0	3	1	0.16678	3.56122	4.0604
NM_178861	ZNF183L1	1	2	2	1	0.27246	2.70492	4.17857
NM_005364	MAGEA8	1	2	3	2	0.44028	1.00461	2.8012
NM_018260	FLJ10891	0	1	1	1	0.53468	0.056067	5.14113
NM_033204	ZNF101	5	0	3	4	0.8654	−1.12073	1.47848
NM_207393	IGFL3	0	1	0	1	0.907	−6.04225	5.56359
NM_000200	HTN3	0	1	0	2	0.98504	−7.36289	4.7631
NM_015703	CGI-96	2	1	0	3	0.99682	−7.76714	4.63335

We discarded RefSeq sequences mapping to multiple Ensembl Genes. “ds,” “ps,” “dn,” and “pn” indicate the number of fixed synonymous sites, the number of polymorphic synonymous sites, the number of fixed non-synonymous sites, and the number of polymorphic non-synonymous sites, respective. “p” indicates whether the gene of interest have an selection intensity (λ = 2Ns) bigger than 0 (neutrality). “u” and “sd” show the estimation of mean and standard deviation of selection intensity. The five genes with *p* smaller than 0.1 were defined as positively selected genes.

### The Excess of New Genes Recruited Into Neocortex Parallels Its Origination

**Figure 5:**
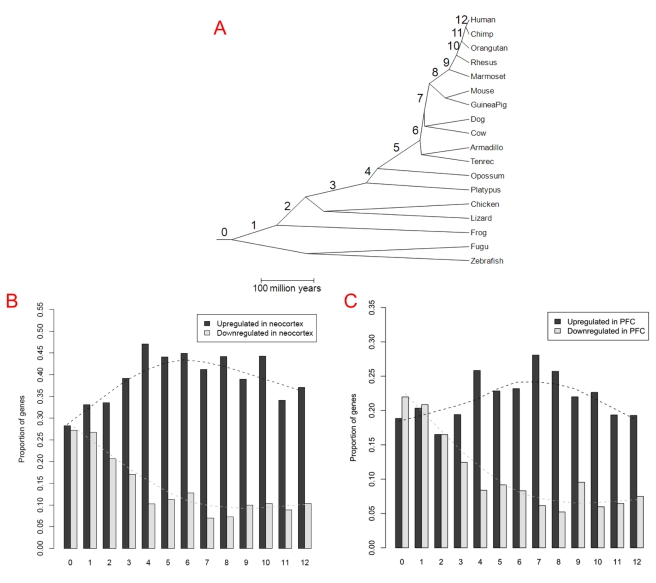
Proportion of genes differentially expressed between neocortex (or PFC) and the non-neocortical regions across different gene ages. (A) The phylogenetic tree together with the branch assignments (0∼12) follows [19]. 0 indicates the oldest gene group, i.e. genes shared by all vertebrates, and branches 8∼12 indicate primate-specific genes, with branch 12 the human-specific lineage. (B) Proportion of genes differentially expressed between neocortex and non-neocortical regions, detected by exon arrays for genes originating in each branch. The dashed line shows the trend fit based on the lowess function of R [56]. (C) Genes with differential expression between PFC and non-neocortical control samples.

If recruitment of new genes into the neocortex was at least partially driven by positive selection for functions in this brain structure, their ages should be correlated with the morphological evolution of neocortex itself. Thus, one prediction is that there would be no excessive recruitment of new genes into the neocortex before it originated. Consistently, the exon array data [4] showed that genes originating after tetrapod and fish split tend to be expressed in the neocortex while only the oldest genes (branch 0, genes shared by all vertebrates) are equally expressed between the neocortex and the non-neocortical regions (Figure 5A, 5B; Table S5). Since genes originating in the tetrapod ancestor (branch 1) already show excessive upregulation in the neocortex (Binomial test *p* = 2×10^−4^ after Bonferroni correction), Figure 5B suggests that the neocortex may have arisen at this time, supporting one viewpoint based on anatomical studies [22]. Such a pattern is consistent with the hourglass model recently observed in zebrafish, where the oldest genes are transcribed in the phylotypic stage (supposedly the stage of ancient evolutionary origin) and younger genes are expressed in the more divergent ontogenic stages [40].

Notably, the timing of new genes expressed in the neocortex shown in Figure 5B could also be explained by the lack of depth in the early branches of the phylogeny. In other words, the excess may actually occur in the common ancestor of vertebrates, but our method based on the vertebrate phylogenetic tree [19] did not detect the hypothesized genes emerging in this period. We took advantage of Ensembl homology annotation [41] and generated a stringent dataset consisting of 879 genes originating in the vertebrate ancestor and 152 genes originating in the chordate ancestor (Materials and Methods). For both groups, there are more genes upregulated in non-neocortical regions (Table S6), confirming that new genes began to be excessively recruited into neocortex since the common ancestor of tetrapods.

**Figure 6:**
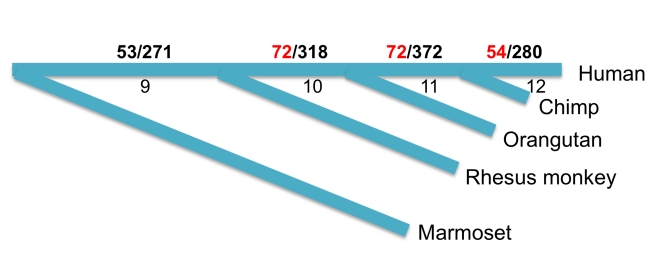
Origination of new genes up-regulated in PFC relative to non-neocortical regions after primate split. Branches 9∼12 follows Figure 5A. The number of genes up-regulated in PFC and the total gene number represented by exon array are shown between “/”. For example, there are 280 human-specific genes, 54 out of which are up-regulated in PFC. In total, there are 198 (72+72+54) genes up-regulated in PFC (marked in RED), which originated along hominoid branches.

Moreover, the anatomical evidence suggests that the PFC is mammal-specific [21]–[22], which provides us a second opportunity to test the temporal correlation. Again, using non-neocortical regions as a control, we traced back to the period when an excess of new genes was recruited into the PFC. Consistent with the anatomical evidence, there was no excessive recruitment of new genes until the ancestral mammals (Figure 5C, branch 3). Such a trend continues into the hominoid lineages with 198 genes upregulated in PFC (Figure 6). Up to 54 of them were human-specific, i.e. they originated after human lineage diverged from the other hominoids. Although these 198 genes have been subject to less experimental investigations, expression of 33 genes in fetal or infant brain was demonstrated by UniGene EST data (Table 3), four of which have been confirmed to encode proteins, as revealed by Pride peptide data [27].

**Table 3:** PFC biased hominoid-specific genes with at least one fetal or infant brain ESTs.

Ensembl v51 ID	Branch	EST#	Description
ENSG00000185984	12	4	solute carrier like
ENSG00000185829	12	3	ADP-ribosylation factor-like protein 17
ENSG00000170161	12	2	Family with sequence similarity 88, member B
ENSG00000205746	12	2	KIAA0220-like protein
ENSG00000154608	12	1	Cep170-like protein
ENSG00000157341	12	1	Putative uncharacterized protein DKFZp547E087
ENSG00000179899	12	1	Putative uncharacterized protein DKFZp686A1782
ENSG00000152117	11	14	Putative uncharacterized protein FLJ41352
ENSG00000183793	11	9	FLJ00322 protein Fragment
ENSG00000196696	11	7	Pyridoxal-dependent decarboxylase domain-containing protein 2 (EC 41.1.-)
ENSG00000100181	11	2	cDNA FLJ42070 fis
ENSG00000170160*	11	2	Coiled-coil domain-containing protein 144A
ENSG00000205534	11	2	Putative uncharacterized SMG1-like protein
ENSG00000132967	11	1	High-mobility group box 1 Fragment
ENSG00000158482	11	1	Putative RUNDC2-like protein 2
ENSG00000180747	11	1	Putative uncharacterized protein LOC641298
ENSG00000182368	11	1	Protein FAM27A/B/C
ENSG00000183444	11	1	MGC72080 protein
ENSG00000183458	11	1	highly similar to Polycystin
ENSG00000196275*	11	1	Transcription factor GTF2IRD2-alpha
ENSG00000213753	11	1	MGC70863 protein
ENSG00000215492	11	1	ROA1_HUMAN Isoform 2
ENSG00000159266	10	6	Pleckstrin homology domain-containing family M member 4
ENSG00000175322*	10	6	Zinc finger protein 519
ENSG00000196267	10	4	Zinc finger protein 836
ENSG00000188933	10	3	Uncharacterized protein ENSP00000344737
ENSG00000196357	10	3	Zinc finger protein 565
ENSG00000183666	10	2	Putative beta-glucuronidase-like protein FLJ75429
ENSG00000174353	10	1	Stromal antigen 3-like
ENSG00000189423	10	1	Proto-oncogene TRE-2-like protein
ENSG00000197054	10	1	Zinc finger protein 763
ENSG00000213413*	10	1	Transmembrane protein PVRIG
ENSG00000214719	10	1	Putative LRRC37B-like protein 2

The four genes with peptide evidence were marked with “*”.

We conducted functional and evolutionary analyses for young genes upregulated in the PFC (Figure 5C) and found similar patterns of GO enrichment and protein evolution as for genes expressed in the developing temporal lobe (Tables S7, S8; Figures S6, S7). For example, out of 13 PFC biased genes covered by [39], five (38%, Table S8) show signals of positive selection, which is significantly higher than old PFC biased genes (14%, FET *p* = 0.03) or the genomic background (15%, FET *p* = 0.03). This similarity might be expected because both the temporal lobe and PFC are part of the neocortex and thus both analyses focused on genes expressed in fetal neocortex. However, finding concordant results from two different parts of the primate neocortex with different technologies strongly suggests that these patterns are robust to methodology and are general across the rapidly evolving neocortex.

## Discussion

### New Genes Are Expressed in the Early Developing Human Brain

Previous analyses of the molecular evolution of the human brain did not find consistent evidence of rapid evolution in the protein-coding genes expressed in the adult human brain [8]–[9]. Faster evolution in the human lineage was not observed at the gene expression level either [2]. However, we noticed that all these analyses were based on the adult brain, just one stage of brain development. It is thus understandable that they were inconclusive as to the understanding of the genetic basis for the evolution of how the brain develops. Our analyses revealed an unexpected pattern: the expression patterns and protein sequences of new genes appear to contribute to the early (fetal and infant) brain development of humans.

This pattern supports the argument that genes formed by duplication and by *de novo* origination could escape pleiotropic constraints [42]. On the other hand, the enrichment of transcription factors in human young genes also suggests the important role of regulation in the development of the human brain [1],[4]–[6]. Our results show that regulatory evolution can occur in both *cis* [5] and *trans*, in the protein sequence of transcription factors [32],[43], and in the creation of new transcription factors through gene duplication. From this aspect, fine-tuning of gene regulation by human-specific genes [44] might underlie many human-specific characteristics and behaviors.

However, we also observed that young genes were associated with diverse functions, ranging from nuclear pore proteins to ribosomal proteins (Table 1). In fact, the striking correspondence of the origination times of the neocortex and PFC with the ages of new genes suggests the functional association of these young genes with the development of these expanding brain structures. Specifically, new genes began to be recruited into neocortex or PFC after their morphological origination (Figure 5B, 5C). The recruitment of young genes into the early developmental stages of neocortex, regardless of the various processes which created these genes (Figures 3, S6), and their accelerated sequence evolution (Figures 4, S6; Tables 2, S8) suggest that the young genes may have evolved new functions as a consequence of positive selection for novel functions in the newly evolved brain structures.

Compared to the early developing brain, the adult brain does not show an increased recruitment of young genes in the primate-specific lineage (Figure S2). Additional expressional data confirmed that young genes were less frequently upregulated in adult neocortex (Figure 2). This result is consistent with a previous study [3] arguing that novel aspects of the human brain are usually manifested in the early development. Thus, the expansion of DUF1220 family expressed in adult brain [20] might be an interesting exception, rather than a rule.

It should be pointed out that our analyses of young genes do not necessarily indicate that old genes are unimportant for human brain evolution. Genome-wide studies that did not consider gene ages have already found that regulation of fetal brain-related genes is evolving [4]–[6]. These observations are actually consistent with our results (Figures 1, 2), since old genes constitute most of the transcriptome of the developing human brain. However, we found that, in contrast to young genes, old genes appear equally expressed in both adult and fetus brains and thus do not have a strong expressional bias toward the fetal brain (Tables S1, S2). This is consistent with the theory that young genes tend to be expressed in evolutionarily young or divergent tissues [40].

### New Genes Are Likely a Target of Positive Selection

Sequence analyses suggest that positive selection could contribute to the evolution of young fetal brain biased genes (Figures 4, S7, Tables 2, S8). This finding expands the cases in which positive selection may act on new genes playing diverse roles such as reproduction [19],[45]–[46], stress response [47]–[48], digestion or metabolism [49]–[51], and mating [52]–[53], in addition to brain development. Thus, new genes may in general be subject to positive selection. For example, in our dataset, even for genes without expression bias, or with expression biased toward the adult brain, McDonald-Kreitman tests [39] demonstrated that 31% (10 out of 32) of new genes show excessive fixation of non-synonymous substitutions, which is significantly higher than the genomic background (FET *p* = 0.02).

However, genetic drift or relaxation of functional constraint may still partially account for the evolution of new genes, especially considering the small effective population size of human [54]. In other words, the evolution of new genes may be often caused by the joint action of drift and positive selection [55].

### Temporal Resolution of New Gene Recruitment into the Developing Brain

We can ask when the fast sequence evolution of new gene proteins happened. We replaced our previous analyses (Figure 4) based on human and chimp alignment with multiple primate genome alignments and inferred the branch-specific *Ka/Ks*. For ancestral branches (branch 10–12 in Figure 5A), all show high *Ka/Ks* with a median of 0.35. Such a result suggests that the fast sequence evolution of fetal brain biased genes may broadly apply for primates.

Notably, our analysis is based on primate- and rodent-specific genes, and transcriptome data from mouse and human. On the one hand, we found 198 human- or hominoid-specific genes which are expressed in PFC of early developing human brain. However, the accelerated origination of new brain development genes we detected may apply for primates in general. Figure 5B/C suggests that a part of this trend may even predate the tetrapod split or mammalian split. Certainly, we cannot be sure whether genes emerging on branch 1 (Figure 5B) indeed have an expression bias toward the amphibian counterpart of the neocortex since our expression analyses use only human and mouse data. Transcriptome data of developing brains in other vertebrates will be valuable in order to determine in which evolutionary period the striking recruitment of new genes began. Finally, even though the excess recruitment of new genes into neocortex begins before the split of tetrapod, it should be pointed out that this trend appears to cease in mouse lineage after its divergence with human since we did not detect a signal in mouse when we focus on rodent-specific genes (Figure 2).

## Materials and Methods

We used MySQL V5.0.45 to organize the data and R V2.10.0 [56] to perform all statistical analyses.

### Gene Dating

We used the gene age data of [19]. Briefly, for Ensembl v51 protein-coding genes [41], we dated their originations by inferring the presence and absence of orthologs along the vertebrate phylogenetic tree based on UCSC syntenic genomic alignment. Compared to methods using only sequence homology between individual genes, our strategy will be more robust in correctly dating fast evolving genes. In other words, although the fast evolving genes may show limited sequence similarity between orthologs, we can generate a syntenic alignment only if their neighboring genes are conserved. In this scenario, we will not mistakenly assign them with younger ages. A comparison between our results and previous efforts revealed that our dating strategy is conservative and we tended to assign older ages to genes [19],[46].

For branch 0 human genes (genes predating the vertebrate split), we took advantage of Ensembl homology annotation [41] and extracted two subsets which consist of genes emerging in the vertebrate ancestor and in the chordate ancestor, respectively. Specifically, the former dataset includes genes that have a one-to-one ortholog in both zebrafish and fugu, but lacking any homolog in the following outgroups: *C. intestinalis*, *C. savignyi*, fruit fly, mosquito, worm, and yeast. The later dataset covers genes which have a one-to-one ortholog in both *C. intestinalis* and *C. savignyi*, but lacking any homolog in fruit fly, mosquito, worm, and yeast.

It is important to note that Ensembl annotation is rapidly changing. Some gene models in v51 (November, 2008) got expired in the latest release v62 (April, 2011). However, even updating our analysis based only on genes retained in v62, the major pattern of young genes biased towards fetal brain relative to old genes (Table S1) continue to holds (FET *p*<2.2×10^−16^, Table S9).

Except elsewhere specified, we defined young genes as primate-specific genes (1,828 genes) in human and rodent-specific genes (3,111 genes) in mouse, respectively, and old genes as those predating the primate and rodent split. Additionally, we use the term “new genes” to describe genes arising as the neocortex originated.

### Gene Annotation

In order to integrate the Bustamante et al. data, we retrieved Ensembl cross-reference information such as Ensembl to EntrezGene [57] mappings with the BioEnsembl [58] based scripts. We used only one-to-one Ensembl ID to Entrez symbol mappings and retained 9,748 genes including 9,682 old genes and 66 young genes. InterPro [30] domain annotations for Ensembl proteins were retrieved with the biomaRt software of Bioconductor system [59].

Gene origination classification and parent/child gene inference follows [19] with one new improvement. We filtered our DNA-level duplicates and retrogene with the retrogene track generated in [60], to ensure the DNA-level duplicates do not overlap with the retrogene track of UCSC, and that our retrogenes are shared by the retrogene track.

We retrieved peptide mapping results from EBI Pride [27] database as of July 2011 with the Bioconductor package, biomaRt [59]. We discarded peptides mapping to multiple Ensembl genes.

### Transcriptional Profiling

Although transcriptional data of the brain are abundant, data covering both the early and late developing brain are not. To our knowledge, there have been no experiments covering different developmental stages across human and mouse. Moreover, human data often focus on one specific subregion of the brain, while mouse data tend to be more general. In order to account for such limitations, we performed extensive transcriptional profiling from several datasets generated by different techniques. A pattern consistent across these datasets would be convincing.

We downloaded EST data from the UniGene database [26], fastq-format RNA-seq data from the SRA database [61], and other raw transcription data from the GEO database [62]. EST data processing including genomic mapping, alignment quality control, and EST-to-gene mapping follows [63]. Only ESTs derived from normal samples were used. We counted a gene as present in a tissue only if it was supported by at least two ESTs. The pattern (Figure 1) remained the same even if we required only one EST.

Microarray data handling included filtering out redundant probes, normalizing, and generating gene-level expression summary, following [19]. Notably, we selected experimental data which used the relative new array designs such as Affymetrix 133 plus 2 or Mouse Genome 430 v2, which provide unique probes for more young genes. Then, since we are mainly interested in the overall difference between early and late brain development, we divided samples into two groups guided by sample clusters generated with functions in Bioconductor packages [59] including dist2, hclust, and levelplot. Finally, we called differential expression with LIMMA software [64] given a false discovery rate (FDR) of 0.05.

For the exon array data of [4], we divided samples into two groups, neocortex (or PFC) and non-neocortical regions (cerebellum, thalamus, striatum, and hippocampus) and then called differential expression with a linear model method [64]. For example, out of 11,819 branch 0 genes, 3,343 (28%) are upregulated in neocortex, while 3,222 (27%) are downregulated.

For RNA-seq data (SRP001119), we calculated gene-level measurement, read count per million per KB (RPMK) following [65]. Specifically, we mapped reads back to the human genome (UCSC hg18) with novoalign v2.05, given its high accuracy [66]. Terminal trimming was enabled to remove possible low-quality bases on the ends of reads. We used the default score difference parameter (“-R 5”), which indicates that the best alignment is about 3-fold more likely than the second best hit. If the best hits failed to pass this parameter, the read would be viewed as mapping to multiple locations and then discarded in the subsequent analyses. This strategy is necessary since young genes are often similar to their parental genes. Then, we ran a second round of mapping against Ensembl transcripts, since novoalign could not handle introns. Multiple-mapping reads were reported in this round since one read often maps to multiple transcripts encoded by the same gene. After mapping reads to genes based on chromosomal coordinates, reads mapping to more than one gene were excluded and read count per gene was calculated. In addition, we generated all possible 32 mers (the length of short reads in SRP001119) based on Ensembl transcript sequences, performed the same mapping process, and counted how many unique 32 mers one gene had. In this way, we generated a modified gene length and finally produced a gene-level RPMK value. Finally, since we are interested in the overall difference between fetus and adult, we pooled six RNA-seq samples into fetus and adult groups and identified genes differentially expressed between these two groups with a generalized likelihood ratio test [67] and a FDR cutoff of 0.05. We did not filter the data with respect to how many unique 32 mers one gene should have except in Figure 3. In order to control for *de novo* genes which may have relatively longer mappable region, duplicated genes with too short a mappable region (<30 bp) were excluded (124 or 0.6% of all genes).

In the case of SAGE data, we downloaded the tag annotation from the SAGEmap database [68], “SAGEmap_Mm_NlaIII_17_best.gz”, and mapped tags to Ensembl genes with unique NCBI Entrez gene symbols. We checked these mappings by searching tag sequences against Ensembl transcripts with novoalign and only kept tag to gene mapping consistent with sequence alignments. After that, we identified differentially expressed genes given a FDR of 0.05 [67].

### Testing Positive Selection

We downloaded 44-way orthologous coding region alignments from the UCSC genome browser [37]. In order to build an human/chimp alignment, we used genes originating before human and chimp split [19] with an alignable region covering more than 100 codons and calculated the nonsynonymous substitution rate (*Ka*) and the synonymous substitution rate (*Ks*) with the CODEML program [69], discarding alignments with less than one synonymous substitution. In testing positive selection, we conducted substitution analyses by taking advantage of the recent divergence of these genes and the available population genetic data [38, 39] when considering the technical inadequacy of the CODEML program [70]. Similarly, we made multiple genomic alignments for the primates, including human, chimp, orangutan, rhesus monkey, or marmoset, and traced how primate-specific genes evolved along the branch leading to human.

## Supporting Information

Figure S1 Proportion of young genes in sub-sampled brain transcriptomes. The *x*- and *y*-axes show the proportion of young genes in the brain transcriptome of mouse and human, respectively. The diagonal line marks where human and mouse brain transcriptomes would have equal contribution of young genes. UniGene consists of 0.9 million (m) ESTs derived from normal human brain samples while only 0.7 m ESTs are derived from normal mouse brain samples. In order to account for this difference, we randomly sampled 0.35 m (half of the mouse sample size) ESTs for both human and mouse for 1,000 times and compared whether the mouse has an equal or bigger proportion of young genes expressed in brain samples. Across all 1,000 replicates, young genes always contribute more in human than in mouse (*p*<0.001).
(TIF)

Figure S2 Young gene contribution in brain transcriptome partitioned by developmental stage. The barplot shows the proportion of young genes out of all genes expressed in adult and fetus brain sample based on EST data, respectively. Sub-sampling as in Figure 1 showed that the fetus brain enrichment in human could not be explained by ascertainment bias (*p*<0.001).
(TIF)

Figure S3 Young gene contribution to transcriptomes of fetal tissues and organs. The barplot shows the proportion of young genes out of all genes expressed in fetus sample of both human and mouse based on EST data. Notably, only brain and heart are significantly different between human and mouse (FET *p* = 2×10^−12^, 0.01, respectively, after multiple test correction). However, the excess in human heart could be accounted for by ascertainment bias (*p* = 0.14).
(TIF)

Figure S4 Proportion of genes associated with enhancers and CTCF binding sites. Enhancer and CTCF annotation were downloaded from [75] and UCSC Encode website, respectively. They were mapped to nearby genes with a cutoff of 100 KB and 10 KB, respectively. Genes were classified into three categories, adult-biased (show higher expression in adult brain), fetus-biased, and unbiased based on the SRA dataset, SRP001119. Gene age (branch) information was from [19].
(TIF)

Figure S5 Chromosomal distribution of young (primate-specific) genes up-regulated in fetal neocortex.
(TIF)

Figure S6 Distribution of genes up- and down-regulated in PFC relative to non-neocortical regions. The pattern is similar to Figure 3 in the main text showing young genes are biased toward PFC expression across all gene origination mechanism.
(TIF)

Figure S7 *Ka/Ks* distribution across different group of genes. The pattern is similar to Figure 4 in the main text with young genes biased expressed toward PFC expression evolving much faster than the other two groups.
(TIF)

Table S1 Statistics of young and old genes with differential expression between different development stages of human brain. The top dataset was obtained from NCBI SRA dataset SRP001199, RNA-sequencing (RNA-Seq) data of fetus and adult human temporal lobe (one part of neocortex). After pooling samples into two groups, fetal and adult samples, we called differential expression with a generalized likelihood ratio test [67] under a false discovery rate (FDR) of 0.05. Fisher's Exact Test (FET) was used to test whether old and young genes follow the same distribution. The middle dataset was obtained from microarray data [71] profiling the superior frontal gyrus (one part of PFC) across different postnatal development stages. We clustered samples into a dendrogram by building a genome-wide expression similarity matrix and divided them into two categories, infant and non-infant brain. Here, samples from humans not older than 1 year old were grouped as infant samples, while the other samples were grouped as non-infant samples. After that, we implemented the LIMMA [64] package to identify differentially expressed genes between two categories under a FDR of 0.05. The bottom dataset [72] profiled dorsolateral prefrontal cortex across different postnatal stages. Similarly, human samples not older than 0.38 years were grouped into the early developing category, while the remaining ones were classified as the late developing category.
(XLS)

Table S2 Statistics of young and old genes with differential expression between different development stages of mouse brain. The top dataset was obtained from fetus and adult cerebral cortex [73] based on SAGE (Serial Analysis of Gene Expression). Analogously, we called differential expression with a generalized likelihood ratio test [67]. Notably, the coverage of genes with SAGE is much lower than that based on RNA-seq due to the much lower sequencing depth of SAGE. The bottom data [74] profiled three postnatal developing time points of the whole brain. Herein, postnatal 0 day samples were classified as the early category, while the other two time points (14 and 56 d) were pooled and classified as the late category.
(XLS)

Table S3 Statistics of young and old genes with differential expression between the adult and fetal brain of humans. Differential expression was detected using RNA-seq data, from SRA dataset SRP001199. Only genes with unique Pride [27] peptide evidence were considered. Again, FET was used to test whether old and young genes follow the same distribution.
(XLS)

Table S4 Expression bias calls based on temporal lobe data. Gene age, expression bias, read count, and *q* value are shown.
(XLS)

Table S5 Differential expression analyses based on exon array data. For fetal brain development data [4], we performed two comparisons: neocortex versus non-neocortical regions (striatum, hippocampus, thalamus, and cerebellum), and PFC versus non-neocortical regions. For each class (neocortex, PFC, and non-neocortical regions), the normalized mean expression intensity across different subregions was shown. Then, the FDR follows for the two comparisons.
(XLS)

Table S6 Statistics of expressional bias for genes originating in the vertebrate and in the chordate ancestor. Notably, there are 10 genes in the former group and one gene in the later group which were not covered by Affymetrix exon array.
(XLS)

Table S7 Over-represented Gene Ontology (GO) terms in PFC biased young genes compared to other young genes. Expression bias was determined using the exon array data [4]. We compared PFC samples and non-neocortical samples (cerebellum, thalamus, striatum, and hippocampus) with LIMMA and identified genes up-regulated in PFC. Only GO terms with a FDR smaller than 0.1 were presented.
(XLS)

Table S8 Selection intensity of young PFC biased genes estimated by McDonald–Kreitman test with Poisson random field [39]. The table convention follows Table 2 in the main text.
(XLS)

Table S9 Statistics of young and old genes with differential expression between different developmental stages of the human temporal lobe. This table is similar to the top panel of Table S1 except that only genes retained in the latest Ensembl v62 were used.
(XLS)
