# Supplementary figures and images for: Accelerated Recruitment of New Brain Development Genes into the Human Genome
Source: PLoS Biol. 2011 Oct 18;9(10):e1001179. doi: 10.1371/journal.pbio.1001179 (PMC3196496; doi:10.1371/journal.pbio.1001179)

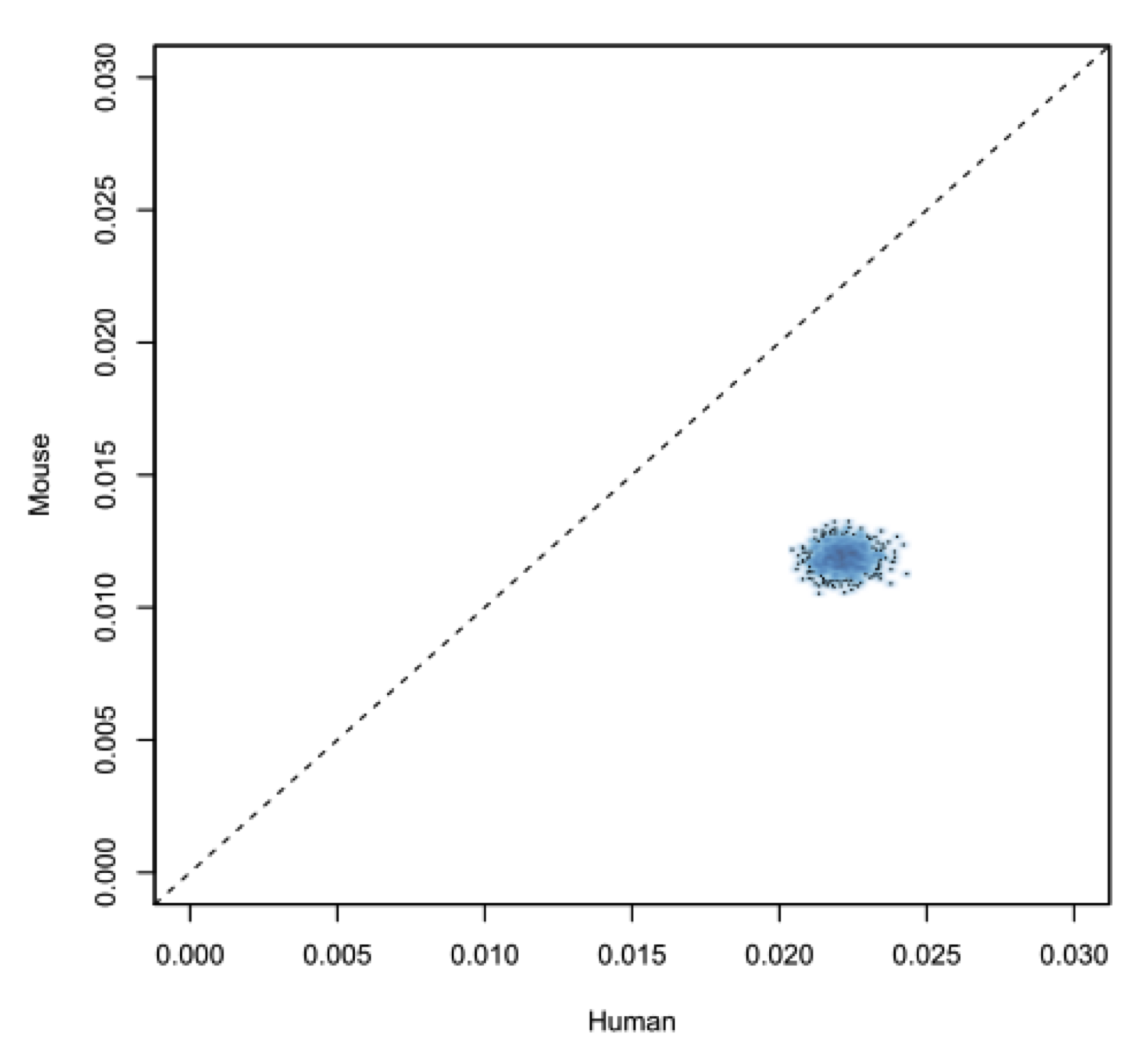

Supplement: Figure S1 — Proportion of young genes in sub-sampled brain transcriptomes. The x- and y-axes show the proportion of young genes in the brain transcriptome of mouse and human, respectively. The diagonal line marks where human and mouse brain transcriptomes would have equal contribution of young genes. UniGene consists of 0.9 million (m) ESTs derived from normal human brain samples while only 0.7 m ESTs are derived from normal mouse brain samples. In order to account for this difference, we randomly sampled 0.35 m (half of the mouse sample size) ESTs for both human and mouse for 1,000 times and compared whether the mouse has an equal or bigger proportion of young genes expressed in brain samples. Across all 1,000 replicates, young genes always contribute more in human than in mouse (p<0.001). (TIF) [file pbio.1001179.s001.tif]

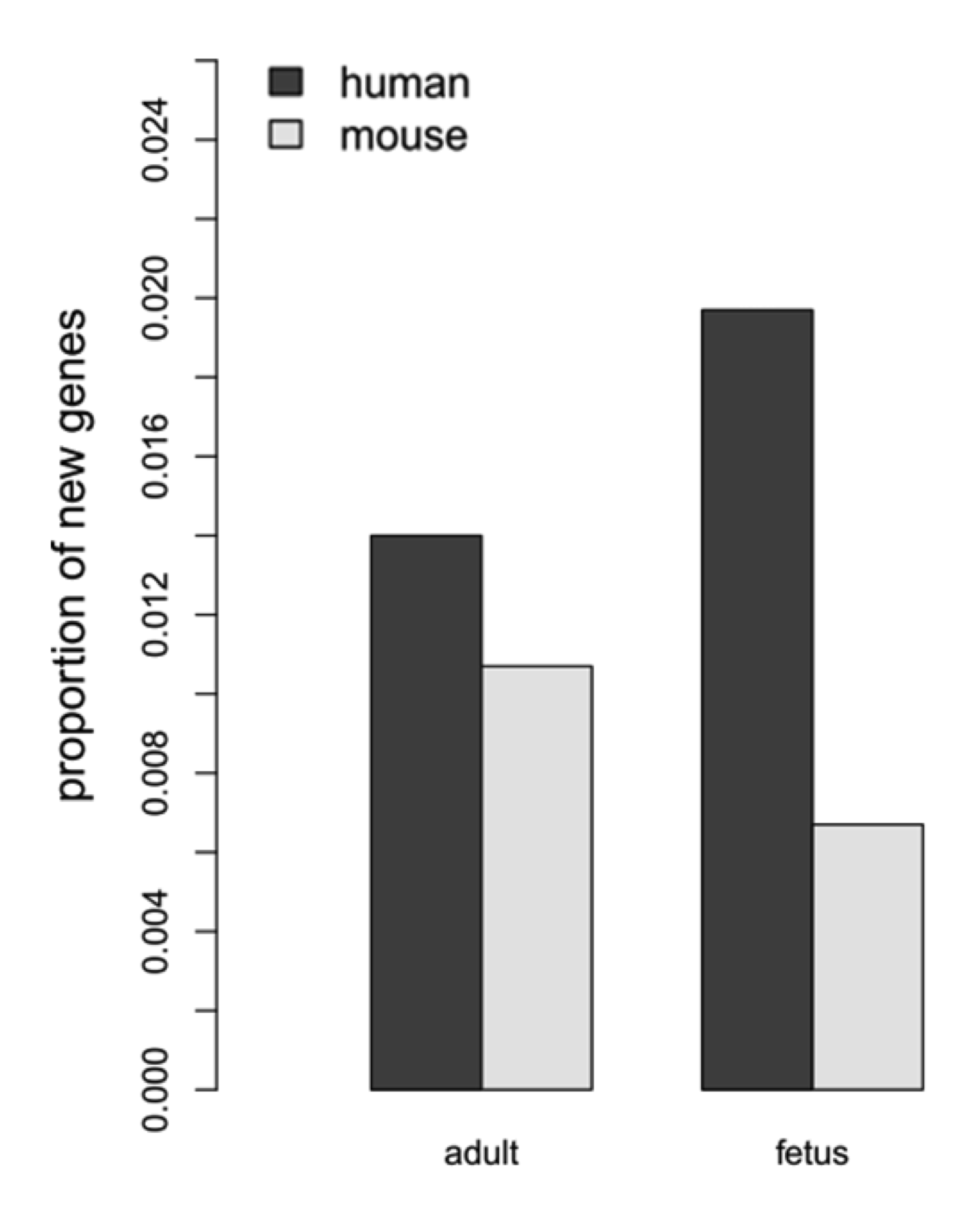

Supplement: Figure S2 — Young gene contribution in brain transcriptome partitioned by developmental stage. The barplot shows the proportion of young genes out of all genes expressed in adult and fetus brain sample based on EST data, respectively. Sub-sampling as in Figure 1 showed that the fetus brain enrichment in human could not be explained by ascertainment bias (p<0.001). (TIF) [file pbio.1001179.s002.tif]

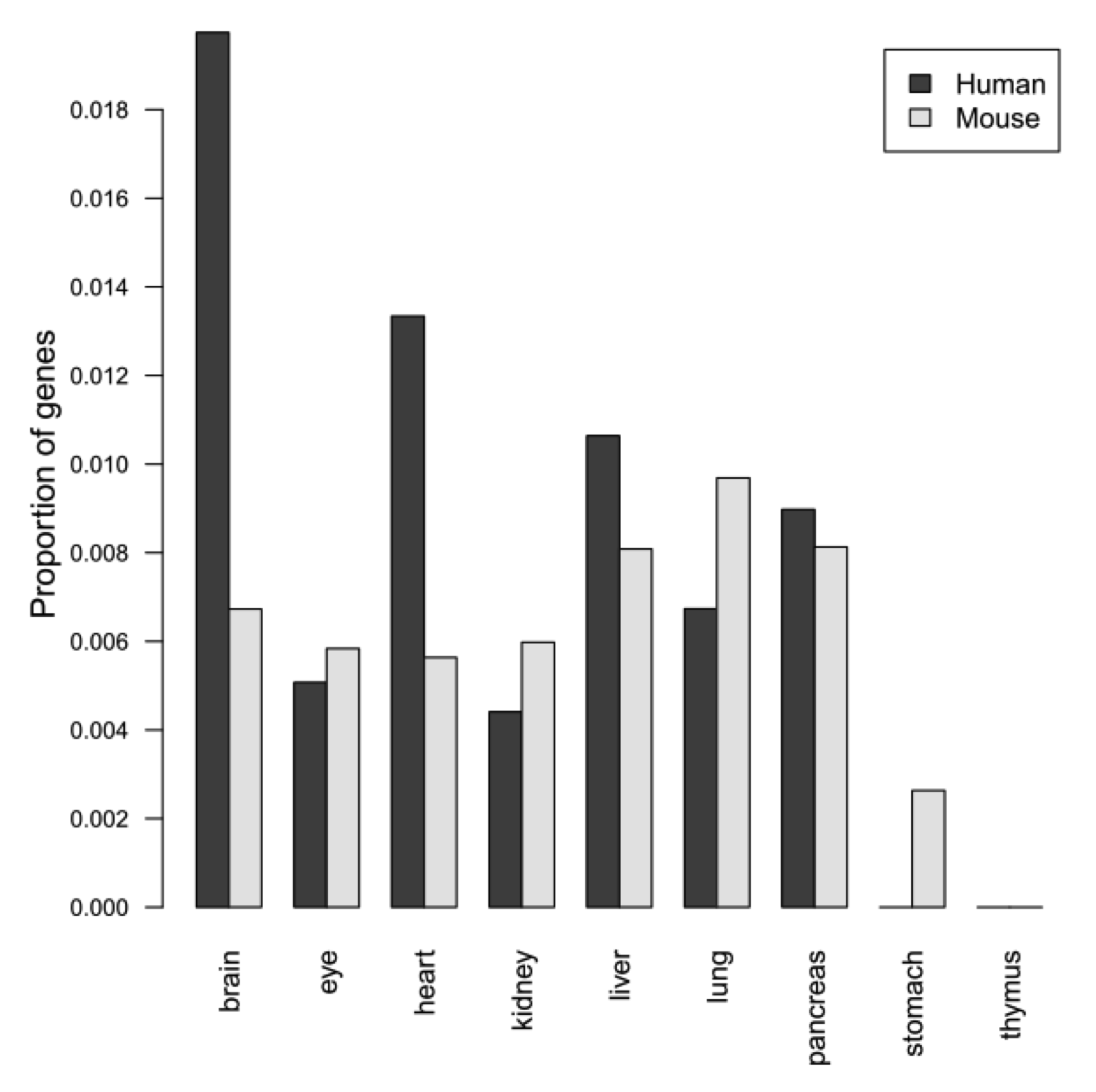

Supplement: Figure S3 — Young gene contribution to transcriptomes of fetal tissues and organs. The barplot shows the proportion of young genes out of all genes expressed in fetus sample of both human and mouse based on EST data. Notably, only brain and heart are significantly different between human and mouse (FET p = 2×10^−12^, 0.01, respectively, after multiple test correction). However, the excess in human heart could be accounted for by ascertainment bias (p = 0.14). (TIF) [file pbio.1001179.s003.tif]

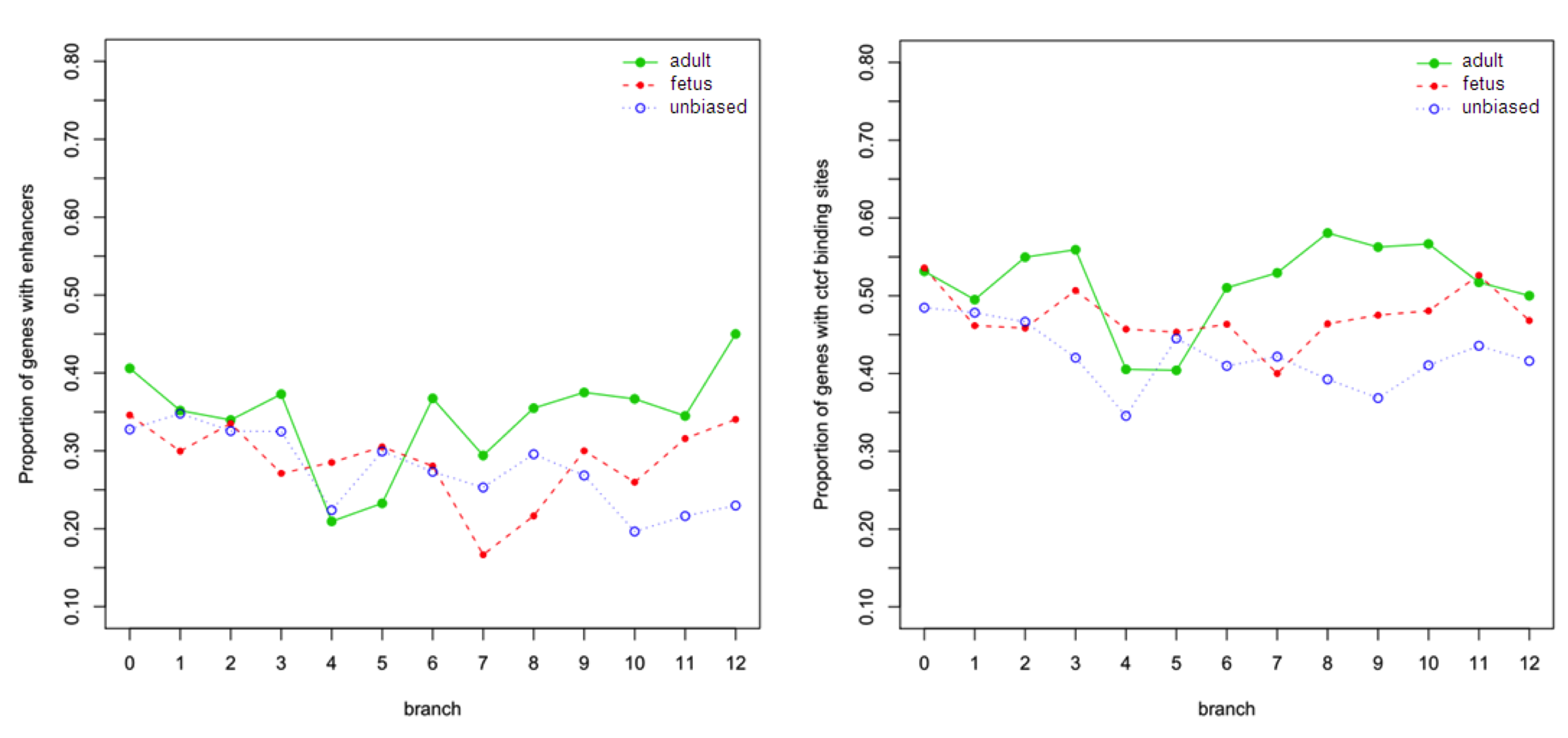

Supplement: Figure S4 — Proportion of genes associated with enhancers and CTCF binding sites. Enhancer and CTCF annotation were downloaded from [75] and UCSC Encode website, respectively. They were mapped to nearby genes with a cutoff of 100 KB and 10 KB, respectively. Genes were classified into three categories, adult-biased (show higher expression in adult brain), fetus-biased, and unbiased based on the SRA dataset, SRP001119. Gene age (branch) information was from [19]. (TIF) [file pbio.1001179.s004.tif]

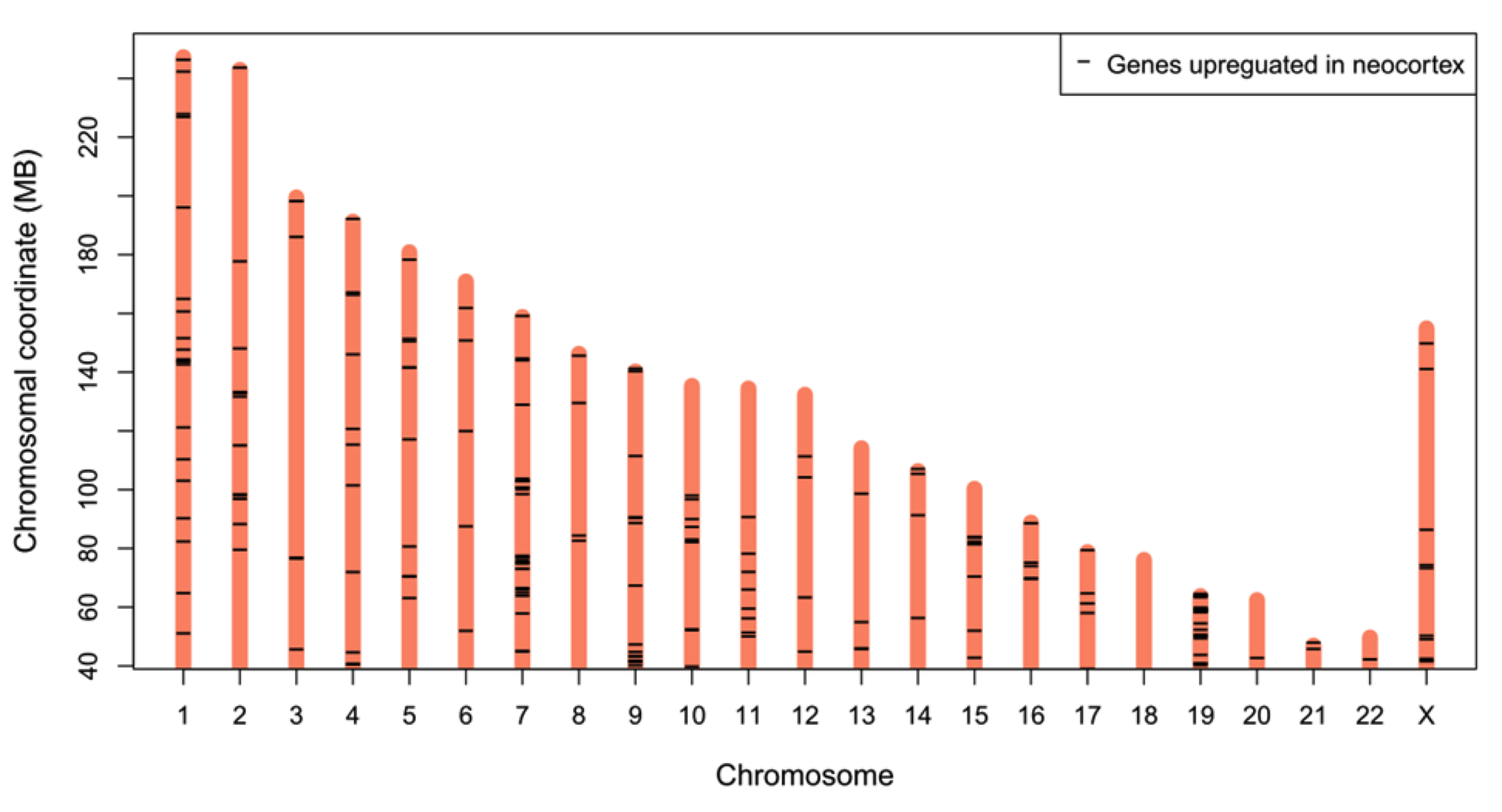

Supplement: Figure S5 — Chromosomal distribution of young (primate-specific) genes up-regulated in fetal neocortex. (TIF) [file pbio.1001179.s005.tif]

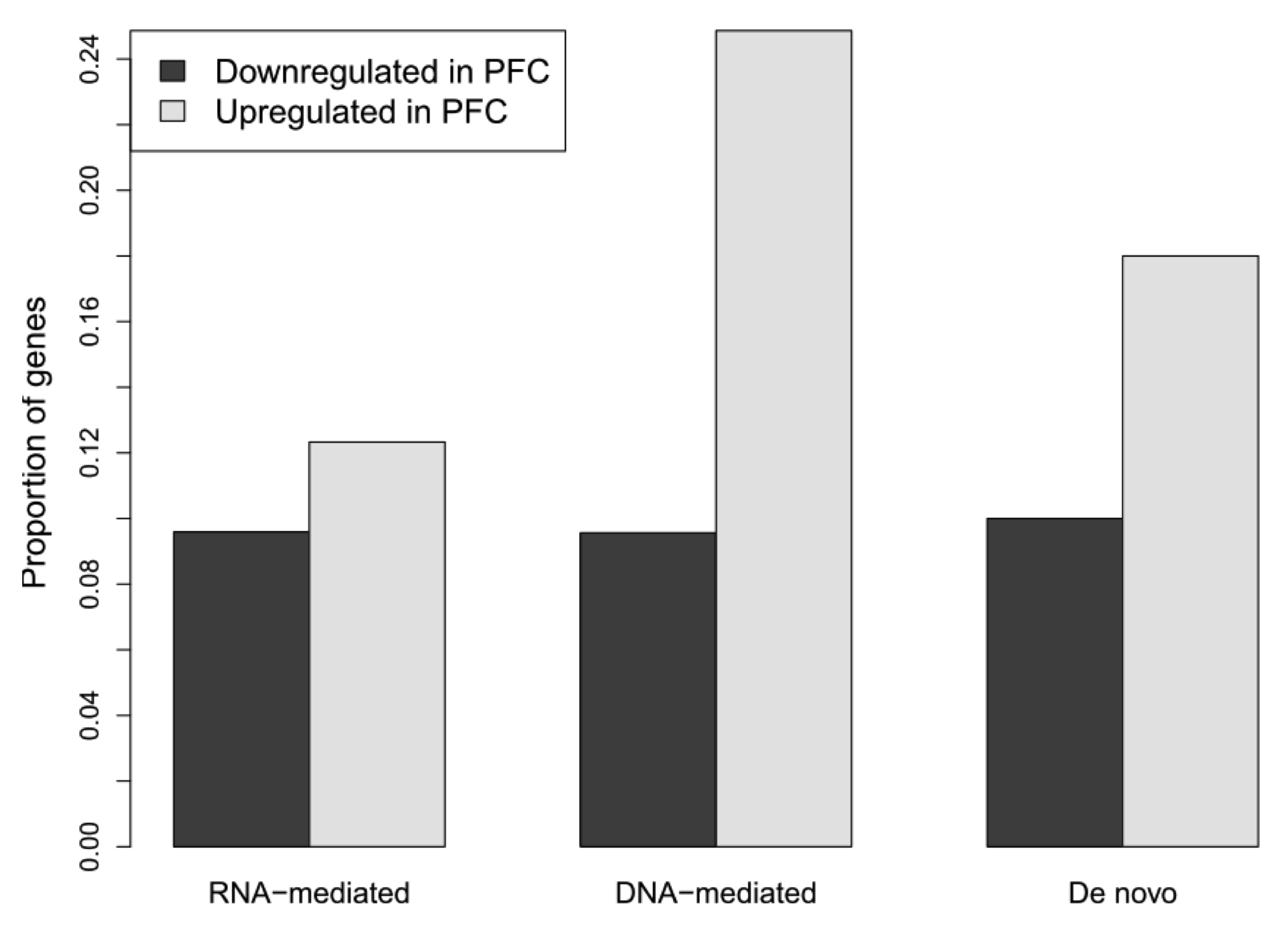

Supplement: Figure S6 — Distribution of genes up- and down-regulated in PFC relative to non-neocortical regions. The pattern is similar to Figure 3 in the main text showing young genes are biased toward PFC expression across all gene origination mechanism. (TIF) [file pbio.1001179.s006.tif]

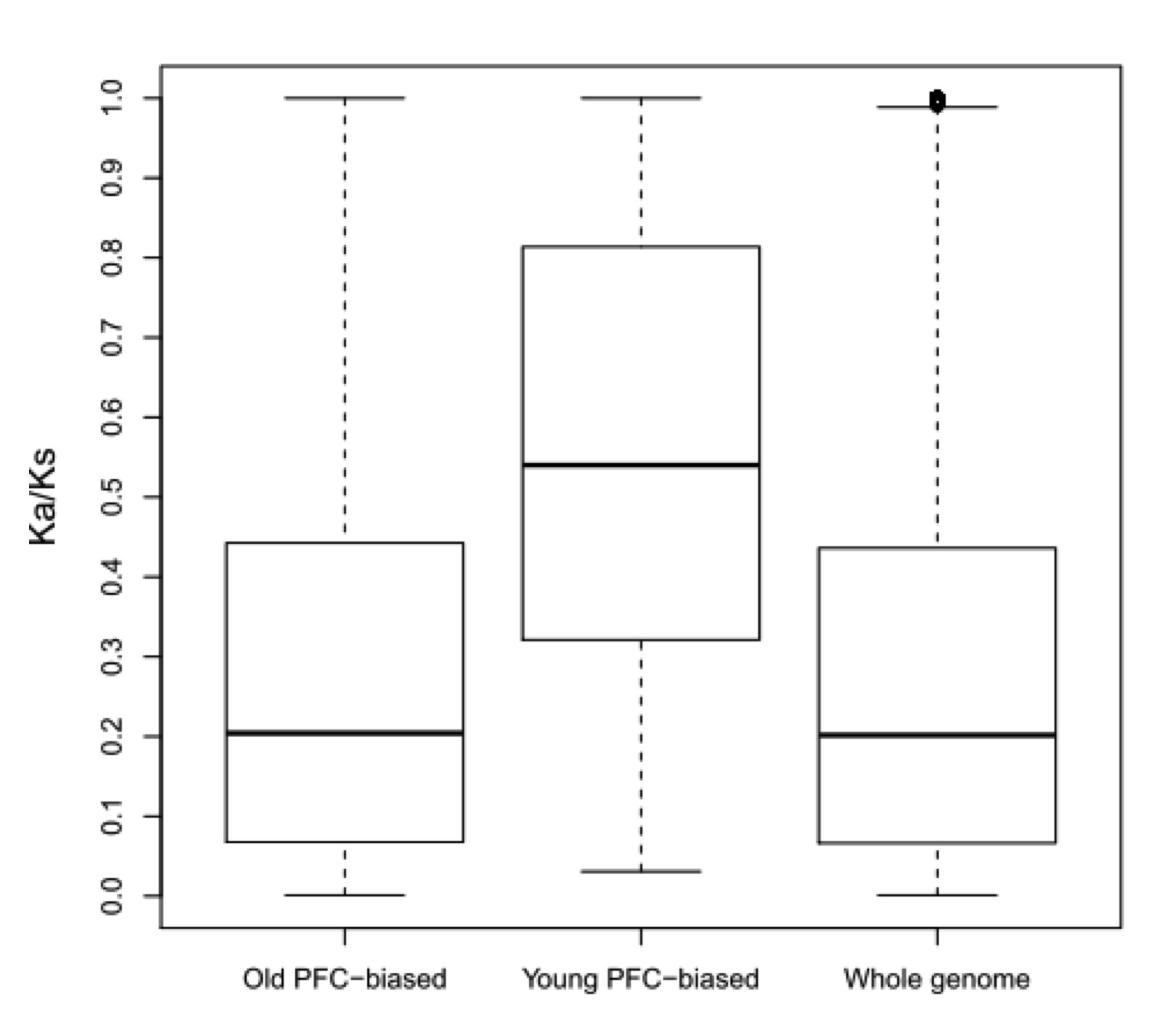

Supplement: Figure S7 — Ka/Ks distribution across different group of genes. The pattern is similar to Figure 4 in the main text with young genes biased expressed toward PFC expression evolving much faster than the other two groups. (TIF) [file pbio.1001179.s007.tif]
